# Lycopene supplementation of maternal and weanling high-fat diets influences adipose tissue development and metabolic outcomes of Sprague-Dawley offspring

**DOI:** 10.1017/jns.2021.91

**Published:** 2021-11-11

**Authors:** Katelyn E. Senkus, Yanqi Zhang, Hui Wang, Libo Tan, Kristi M. Crowe-White

**Affiliations:** Department of Human Nutrition, The University of Alabama, Tuscaloosa, AL, USA

**Keywords:** Lycopene, High-fat diet, Brown adipose tissue, Maternal obesity, Redox status, Metabolic health, AT, adipose tissue, BAT, brown adipose tissue, BW, body weight, HFD, high-fat diet, HFDL, HFD supplemented with 1% lycopene, NFD, normal fat diet, WAT, white adipose tissue, UCP1, uncoupling protein 1, AI, Adiposity Index, MDA, Malondialdehyde, AC, Antioxidant Capacity

## Abstract

Dietary patterns high in fat contribute to the onset of cardiometabolic disease through the accrual of adipose tissue (AT). Lycopene, a carotenoid shown to exert multiple health benefits, may disrupt these metabolic perturbations. The purpose of the present study was to evaluate AT development and obesity-associated metabolic outcomes in the neonate and weanling offspring of Sprague-Dawley mothers fed a high-fat diet (HFD = 50 % fat) with and without lycopene supplementation. Sprague-Dawley rats consumed either a normal fat diet (NFD; 25 % fat) or HFD throughout gestation. Upon delivery, half of HFD mothers were transitioned to an HFD supplemented with 1 % lycopene (HFDL). At postnatal day 14 (P14), P25, and P35, pups were euthanised, body weight was recorded, and visceral white AT (WAT) and brown AT (BAT) mass were determined. Serum redox status, adipokines, glucose and inflammatory biomarkers were evaluated, as well as BAT mRNA expression of uncoupling protein 1 (UCP1). The HFD was effective in inducing weight gain as evident by significantly greater BW and WAT in the HFD group compared to the NFD group across all time points. Compared to HFD, the HFDL group exhibited significantly greater BAT with concomitant reductions in WAT mass, serum lipid peroxides and serum glucose. No significant differences were observed in serum adipokines, inflammatory markers or UCP1 expression despite the aforementioned alterations in AT development. Results suggest that dietary lycopene supplementation may influence metabolic outcomes during the weaning and post-weaning periods. Additional research is warranted to elucidate molecular mechanisms by which lycopene influences AT biology.

## Graphical abstract

**Figure:**
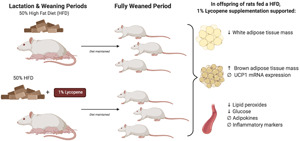

## Introduction

With the rising prevalence of obesity, it is no surprise that approximately 50 % of women enter pregnancy while overweight or obese^(1)^. The increased risk for adverse pregnancy outcomes is further compounded by the likelihood of these individuals to exceed gestational weight gain recommendations. Collectively, this excess adiposity during pregnancy has both short- and long-term implications for the developing foetus including increased birth weight and cardiometabolic disease risk^(2,3)^.

Maternal obesity and excess pregnancy weight gain promote increased white adipose tissue (WAT) stores in offspring^(3)^. Although previously considered benign, it is now well established that WAT is a metabolically dynamic organ^(4)^. The endocrine function of WAT contributes to cardiometabolic disease onset through its disruption of metabolic homeostasis, including redox and inflammatory imbalance, as well as alterations in adipokine secretion and glucose dynamics. Humans also possess variable amounts of the thermogenic brown adipose tissue (BAT). While WAT and BAT share general properties, including their heterogeneous compositions and high levels of vascularisation and innervation, they are distinct in their metabolic regulation and, thus, influence on human health^(5)^. BAT is unique with its dense mitochondrial make-up and expression of uncoupling protein 1 (UCP1), enabling it to uncouple oxidative phosphorylation and dissipate energy as heat through a process known as adaptive thermogenesis^(6)^. Accordingly, these thermogenic cells have a high nutrient consumption paired with an elevated energy expenditure, resulting in increased whole-body energy expenditure with the potential for reduced body weight (BW) and WAT accrual. Taken collectively, the metabolic perturbations of an obese pregnancy may be counterbalanced by the metabolic benefits of BAT.

The development of BAT is heightened in the foetus because of its role in the physiological adaptations that accompany the transition from an intra- to extrauterine environment^(7,8)^. For example, large depots of BAT present in the interscapular region of newborns support the maintenance of core body temperature. BAT present at birth can increase in mass via hyperplasia or hypertrophy throughout early life, owing to its unique thermogenic genetic signature that is susceptible to epigenetic programming. However, without the appropriate stimuli to promote maintenance and/or initiate continued development, BAT stores will gradually decline with ageing. Previous research suggests that a greater BAT depot at birth is associated with reduced body fat gain during early life, equating to an attenuated child and adulthood obesity risk^(9)^. As such, investigating nutritional strategies to increase BAT development in the neonate and to preserve this tissue throughout development is critical.

Carotenoids are a class of lipid-soluble compounds found predominantly in fruits and vegetables^(10)^. A subset of carotenoids has been demonstrated to metabolically activate adaptive thermogenesis. For example, β-carotene, α-carotene and lutein dose-dependently upregulate UCP1 expression in primary mouse brown adipocytes^(11)^. Previous research also suggests that the vitamin A derivative retinoic acid increases UCP1 expression in BAT and augments its thermogenic capabilities in both *in vitro* cell cultures and *in vivo* animal models^(12,13)^. Acknowledging the emerging role of carotenoids in adaptive thermogenesis, continued investigation of other compounds within this family is warranted.

Lycopene, a potent antioxidant with singlet oxygen quenching abilities, is a carotenoid found most concentrated in tomatoes, tomato products and watermelon^(14)^. This carotenoid has been shown to exert multiple health benefits, including antioxidant and anti-inflammatory functionality, as well as improvements in glucose dynamics and adiposity^(15–18)^. It is unknown, however, whether lycopene metabolically activates adaptive thermogenesis like other carotenoids and, thus, disrupts the ensuing dysfunction of an excessive energy burden during pregnancy. Additionally, the antioxidant properties of lycopene are well established with its direct ingestion; yet, its ability to impact metabolic health when consumed indirectly during critical phases of development remains to be elucidated. As such, the aim of the present study was to determine the influence of lycopene supplementation on adipose tissue (AT) development and a range of obesity-associated metabolic outcomes, including serum redox status, adipokines, glucose and inflammatory cytokines, in the neonate and weanling offspring of Sprague-Dawley mothers fed a high-fat diet. It was hypothesised that lycopene supplementation would beneficially alter WAT and BAT development in offspring while concurrently improving circulating measures of metabolic health.

## Materials and methods

### Experimental animals and study design

**Fig. 1.**
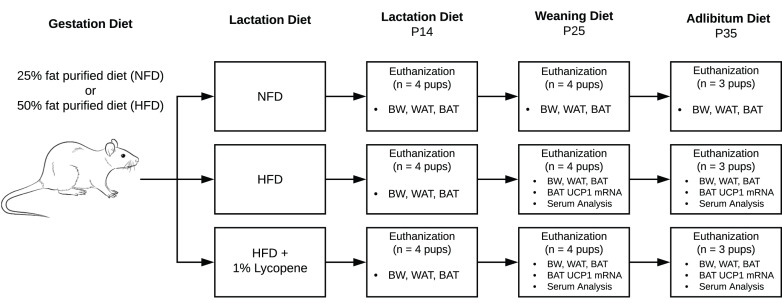
Flow of experimental procedures. Timed-pregnant Sprague-Dawley rats arrived on their second day of gestation and were randomised to a 25 % NFD or a 50 % HFD for the remainder of gestation. Upon delivery, half of the HFD group mothers were transitioned to an HFD supplemented with 1 % lycopene. The others continued NFD or HFD without lycopene supplementation through P25. Beginning at P25, the remaining pups were directly fed the diets of their respective mothers until P35. At P14, P25, and P35, pups from each litter were euthanised, and the following outcome measures were assessed: BW (all), visceral WAT mass (all), interscapular BAT mass (all), UCP1 mRNA expression in BAT (P25 and P35), serum redox status (P25 and P35), serum adipokines, glucose, and inflammatory biomarkers (P25). BAT, brown adipose tissue; BW, body weight; HFD, high-fat purified diet (50 %); NFD, normal fat purified diet (25 %); P14, P25, and P35, postnatal day 14, 25, and 35, respectively; UCP1 mRNA, uncoupling protein 1 messenger RNA; WAT, white adipose tissue.

Timed-pregnant Sprague-Dawley rats were purchased from Charles River (Wilmington, MA, USA). Animals arrived on their second day of gestation and were individually housed under controlled conditions (23 ± 1 °C, 12-12 h light-dark cycle) with free access to food and water. All procedures were compliant with the Guide for the Care and Use of Laboratory Animals and approved by the Institutional Animal Care and Use Committee (IACUC) of The University of Alabama (Tuscaloosa, AL, USA) (Fig. 1).

Following a 3-d acclimation period, animals were randomised to a 25 % kcal from fat purified diet (normal fat diet, NFD) or a 50 % kcal from fat purified diet (high-fat diet, HFD) for the remainder of gestation (Research Diets, New Brunswick, NJ, USA) (Table 1). Animals were monitored daily, and the day of parturition was defined as postnatal day 0 (P0). Upon delivery of pups, half of the HFD group mothers were transitioned to an HFD supplemented with 1 % lycopene (HFDL; LycoVit® dispersion 10 %, BASF North America, Florham Park, NJ, USA). The others continued NFD or HFD without lycopene supplementation through P25. This dose was determined by previous studies showing no evidence of toxicity in rats that were supplemented with 1 % lycopene for up to 90 d^(19)^. Beginning at P25, the pups were fully weaned, transferred to individual cages, and directly fed the diets of their respective mothers until study completion on P35. Diets were replaced daily throughout the study, and food consumption was determined at the time of change. The amount of lycopene ingested was calculated from daily food consumption.

**Table 1.** Diet composition

Ingredient	Normal fat diet^a^		High-fat diet^b^	
	g	kcal	g	kcal
Casein	200	800	200	800
L-Cystine	3	12	3	12
Corn starch	353⋅8	1415	101⋅2	405
Maltodextrin	125	500	125	500
Sucrose	68⋅8	275	68⋅8	275
Cellulose	50	0	50	0
Soyabean oil	25	225	25	225
Lard	87⋅7	789	200	1800
Mineral mix, S10026	10	0	10	0
Dicalcium phosphate	13	0	13	0
Calcium carbonate	5⋅5	0	5⋅5	0
Potassium citrate	16⋅5	0	16⋅5	0
Vitamin mix, V10001	10	40	10	40
Choline bitartrate	2	0	2	0
Food colour	0⋅05	0	0⋅05	0
Total				
	g%	kcal%	g%	kcal%
Fat	12	25	27	50
Carbohydrate	57	55	37	30
Protein	21	20	24	20
Total	–	100	–	100
kcal/g	4⋅2	–	4⋅9	–

Diet composition for Sprague-Dawley rats fed normal or high-fat purified diets. Formulation details are provided in grams, g, and kilocalories, kcal.

a Research diets (rodent diet with 25 % kcal fat, D18100206).

b Research diets (rodent diet with 50 % kcal fat, D18100207).

### Collection and preparation of samples

At P14, P25 and P35, pups from each group were euthanised via carbon dioxide inhalation and cervical dislocation with BW recorded (P14 = four pups; P25 = four pups; P35 = three pups). Blood collected from the vena cava was centrifuged, and the obtained serum was stored at −80 °C until analysis. Visceral WAT and interscapular BAT were harvested, weighed, and snap-frozen in liquid nitrogen for storage at −80 °C. The adiposity index (AI) was determined as the ratio of visceral WAT mass to BW at all time points^(20)^.

### Serum and tissue analysis

#### Serum lycopene analysis

All reagents and solvents for methods described herein were purchased through Sigma-Aldrich (St. Louis, MO, USA) and VWR (Atlanta, GA, USA). Serum lycopene extraction was conducted according to a previously validated procedure^(21)^. Chromatographic separation was carried out on serum samples collected from P14, P25, and P35 using an ACQUITY ultra-high performance liquid chromatography system with a photodiode array detector and ACQUITY BEH Shield RP18 2⋅1 × 100 mm, 1⋅7 μm (Waters, Milford, MA, USA) according to a previously described method for fat-soluble micronutrients^(22)^. Lycopene and β-Apo-8′-carotenal were sourced from Sigma-Aldrich, and β-Apo-8′-carotenal served as the internal standard. The limit of quantitation for lycopene was 0⋅039 μmol/l.

#### Serum redox status

##### Oxidative stress

Malondialdehyde (MDA), a product of lipid peroxidation, is a biomarker of oxidative stress. Serum lipid peroxides were quantified at P25 and P35 according to the thiobarbituric acid reactive substances assay as previously described^(23)^. Results are expressed as mM MDA.

##### Antioxidant capacity

Serum was deproteinated using methanol/acetonitrile/acetone (1:1:1, v/v/v) added to samples in a ratio of 1:4 (v/v)^(24)^. This method enables detection of small molecular weight antioxidants (<6 kDa). Lipophilic antioxidant capacity (AC) was measured at P25 and P35 using the oxygen radical absorbance capacity assay on a FLUOstar Optima plate reader (BMG Labtech, Cary, NC, USA)^(25)^. The compound 2,2-azobis(2-amidino-propane) dihydrochloride was used as the peroxyl radical generator and Trolox, a water-soluble analogue of vitamin E, served as the reference antioxidant standard. Results are expressed as μM Trolox equivalents.

#### Serum adipokines, glucose and inflammatory biomarkers

Adiponectin and leptin were quantified using enzyme-linked immunosorbent assays (Millipore Rat Adiponectin ELISA and Millipore Rat Leptin ELISA, respectively, Billerica, MA, USA). Minimum sensitives for adiponectin and leptin assays were 1⋅6 μg/mL and 0⋅2 ng/mL, respectively. Glucose was measured by enzymatic methods using an automated analyser (Stanbio Sirus analyser, Boerne, TX, USA) with a 2 mg/dl minimum sensitivity. Concentrations of inflammatory biomarkers, including interleukin-10 (IL-10), interleukin-6 (IL-6) and tumour necrosis factor-α (TNF-α), were assessed using Meso Scale Discovery MESO QuickPlex SQ 120 imager (Rockville, MD, USA). Minimum sensitivities were 2⋅74, 33⋅32 and 0⋅64 pg/mL for IL-10, IL-6 and TNF-α, respectively. Serum samples were analysed for adipokines, glucose and inflammatory biomarkers at P25 only.

#### Uncoupling protein 1 messenger RNA expression in BAT

Total RNA was isolated from BAT using an RNA extraction kit (Trizol, Invitrogen, Waltham, MA, USA). RNA was reverse-transcribed into cDNA using qScript® cDNA synthesis kit (QuantaBio, Beverly, MA, USA), and mRNA expression was quantified by real-time qPCR analysis at P25 and P35. The rat primer sequence designed to detect uncoupling protein 1 messenger RNA (UCP1 mRNA) expression was (NM_012682.2), 5′-AGAAGGATTGCCGAAACTGTAC-3′ (forward primer) and 5′-AGATCTTGCTTCCCAAAGAGG-3′ (reverse primer). β-actin served as the housekeeping gene. Relative gene expression was calculated using the 2^−ΔΔCT^ method^(26)^.

### Statistical analysis

Since distinction in adiposity was integral to the study design, a power calculation based upon expected differences in AT accrual was performed to determine sample size. Power analysis generated a sample of *n* 3 pups/group at each time point for 80 % power at an α level of 0⋅05. Data are presented as mean  (standard deviation). Mean differences between groups were evaluated using *t*-tests. Associations between outcome measures were also assessed using Pearson's correlation coefficient. Statistical significance was defined as *P* < 0⋅05. All analyses were performed using SPSS version 25 (SPSS Inc., Chicago, IL, USA).

## Results

### Dietary and lycopene intake

#### Diet-induced obesity model: dietary fat intake

Beginning on day 5 of gestation, all mothers were fed either an NFD or an HFD without lycopene supplementation. Mothers maintained the assigned NFD or HFD throughout study duration. If the mother was randomised to the HFDL group, lycopene supplementation began in conjunction with the HFD upon delivery (P0). Although a greater average dietary intake was observed in the NFD group as compared to the mothers consuming an HFD (NFD: 21⋅95 (2⋅95) g/d, HFD: 16⋅89 (2⋅81) g/d, HFDL: 15⋅40 (3⋅97) g/d), the mothers consuming the HFD maintained a higher fat consumption throughout gestation (NFD: 2⋅63 (0⋅35) g/d, HFD: 4⋅56 (0⋅76) g/d, HFDL: 4⋅16 (0⋅74) g/d). During the lactation and weaning periods, fat consumption remained consistently higher among mothers consuming an HFD (P14 – NFD: 4⋅69 (1⋅47) g/d, HFD: 9⋅98 (3⋅20) g/d, HFDL: 9⋅70 (2⋅12) g/d; P25 – NFD: 6⋅81 (0⋅71) g/d, HFD: 17⋅18 (3⋅95) g/d, HFDL: 14⋅44 (3⋅40) g/d). This pattern of dietary fat intake was also observed once pups were transitioned to *ad libitum* diets of their respective mothers through P35 (NFD: 1⋅32 (0⋅09) g/d, HFD: 3⋅14 (0⋅53) g/d, HFDL: 3⋅24 (0⋅36) g/d).

#### HFD *v*. HFDL groups: total dietary and lycopene intake

At P0, half of the mothers consuming an HFD began consuming 1 % lycopene-supplemented diets. Mothers from both the HFD and HFDL groups maintained a comparable dietary intake that steadily increased between necropsy time points (P14 – HFD: 36⋅95 (11⋅87) g/d, HFDL: 35⋅94 (7⋅85) g/d; P25 – HFD: 63⋅62 (14⋅64) g/d, HFDL: 53⋅49 (12⋅60) g/d) (Table 2). Beginning at P25, the remaining pups were directly fed the diets of their respective mothers. No significant differences in dietary intake were observed between groups (HFD: 11⋅64 (1⋅94) g/d *v*. HFDL: 11⋅99 (1⋅33) g/d). Dietary lycopene intake levels for mothers of the HFDL group were 0⋅36 (0⋅08) g/d and 0⋅53 (0⋅13) g/d at P14 and P25, respectively. Dietary lycopene intake level for respective pups was 0⋅12 (0⋅01) g/d at P35.

**Table 2.** Dietary intake

	P14	P25	P35
Total dietary intake (g/d)			
NFD	39⋅11 (12⋅23)	56⋅76 (5⋅90)	11⋅04 (0⋅71)
HFD	36⋅95 (11⋅87)	63⋅62 (14⋅64)	11⋅64 (1⋅94)
HFDL	35⋅94 (7⋅85)	53⋅49 (12⋅60)	11⋅99 (1⋅33)
Energy intake (kcal/d)			
NFD	164⋅26 (51⋅37)	238⋅39 (24⋅78)	46⋅37 (2⋅97)
HFD	181⋅06 (58⋅16)	311⋅74 (71⋅74)	57⋅02 (9⋅52)
HFDL	176⋅11 (38⋅47)	262⋅1 (61⋅74)	58⋅77 (6⋅52)
Dietary fat intake (g/d)			
NFD	4⋅69 (1⋅47)	6⋅81 (0⋅71)	1⋅32 (0⋅85)
HFD	9⋅98 (3⋅2)	17⋅18 (3⋅95)	3⋅14 (0⋅53)
HFDL	9⋅70 (2⋅12)	14⋅44 (3⋅40)	3⋅24 (0⋅36)
Lycopene intake (g/d)			
NFD	–	–	–
HFD	–	–	–
HFDL	0⋅36 (0⋅08)	0⋅53 (0⋅13)	0⋅12 (0⋅01)

NFD, normal fat purified diet (25 %); HFD, high-fat purified diet (50 %); HFDL, high-fat purified diet (50 %) with 1 % lycopene supplementation; P14, P25, and P35, postnatal day 14, 25, and 35, respectively.

Total dietary intake (g/d), energy intake (kcal/d) and lycopene (g/d) daily intake in Sprague-Dawley rats fed a normal fat diet, high-fat diet or high-fat diet with 1 % lycopene supplementation. Each value represents the mean (standard deviation). P14 and P25 represent maternal dietary consumption. Beginning at P25, the remaining pups were fully weaned and directly fed the diets of their respective mothers; therefore, P35 represents offspring dietary consumption.

### Circulating lycopene

Lycopene peaks were observed at 450 nm in serum of the HFDL group at all time points. Similar peaks were not present in the HFD only group. Although lycopene was transmitted into the circulation of HFDL pups, serum concentrations remained below the level of quantitation but above the level of detection across time points.

### Body weight and adipose tissue

#### NFD *v*. HFD group: assessing diet-induced obesity

The HFD was effective in inducing weight gain as evidenced by increases in BW and WAT in HFD pups not receiving lycopene supplementation compared to pups from the NFD litter across time points. BW of pups was significantly higher in the HFD group at P14 and P25 (13⋅5 % greater, *P* = 0⋅029; 33⋅3 % greater, *P* < 0⋅001, respectively). Notably, pups reared by mothers consuming the HFD exhibited a WAT mass that was 8⋅1, 3⋅5 and 2⋅0 times greater than pups from the NFD litter at P14 (*P* < 0⋅001), P25 (*P* < 0⋅001) and P35 (*P* = 0⋅003), respectively. Acknowledging that the aim of this present study was to evaluate the influence of lycopene supplementation in a diet-induced obesity model during the pre- and post-weaning periods, the aforementioned results confirm the achievement of diet-induced obesity. As such, the remaining results will highlight only the HFD and HFDL groups.

#### BW and AT development

**Fig. 2.**
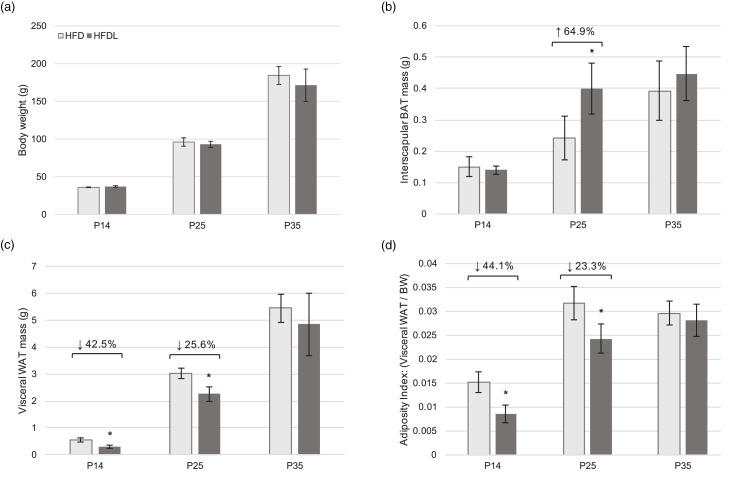
Body weight and adipose tissue development in offspring. Body weight (a), visceral white adipose tissue mass (b), interscapular BAT mass (c), and adiposity index (d) of offspring of Sprague-Dawley mothers fed a high-fat diet with or without lycopene supplementation. Bars represent the mean ± standard deviation. *Indicates *P* < 0⋅05 between-group difference. BAT, brown adipose tissue; BW, body weight; HFD, high-fat purified diet (50 %); HFDL, high-fat purified diet (50 %) with 1 % lycopene supplementation; P14, P25, and P35, postnatal day 14, 25, and 35, respectively; WAT, white adipose tissue.

No significant differences in BW were observed between pups from the HFD and HFDL litters across all time points (Fig. 2(a)). However, beginning at P14, WAT was 42⋅5 % lower (*P* = 0⋅003) in pups reared by mothers consuming HFDL compared to the HFD group (Fig. 2(b)). At P25, significant decreases in WAT (25⋅6 % lower, *P* = 0⋅004) were also observed concomitantly with significant increases in BAT (64⋅9 % increase, *P* = 0⋅025) in pups reared by mothers consuming HFDL compared to the HFD group not receiving lycopene (Fig. 2(b) and (c)). Albeit non-significant, WAT in the HFDL pups remained lower, while BAT remained higher through P35. Additionally, the AI was significantly lower in pups from the HFDL group at P14 and P25 (44⋅1 % lower, *P* < 0⋅05; 23⋅3 % lower, *P* < 0⋅05, respectively) (Fig. 2(d)).

#### UCP1 mRNA expression in BAT

No significant between-group differences were observed in the BAT mRNA expression of UCP1 in pups from the HFD and HFDL litters at P25 or P35. Similarly, there were no significant within-group changes in expression from P25 to P35 for either the HFD or HFDL groups.

### Obesity-associated metabolic outcomes

#### Redox status

**Fig. 3.**
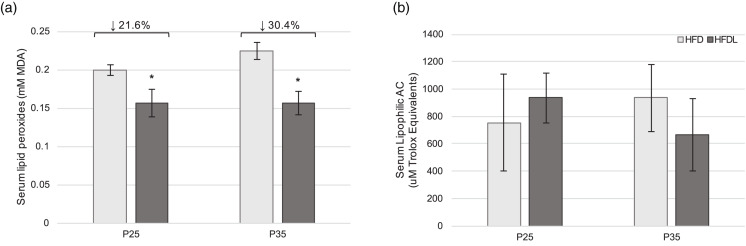
Serum redox status in offspring. Serum lipid peroxides (a) and lipophilic antioxidant capacity (b) in offspring of Sprague-Dawley mothers fed a high-fat diet with or without lycopene supplementation. Bars represent mean ± standard deviation. *Indicates *P* < 0⋅05 between-group difference. AC, antioxidant capacity; HFD, high-fat purified diet (50 %); HFDL, high-fat purified diet (50 %) with 1 % lycopene supplementation; MDA, malondialdehyde; P25 and P35, postnatal day 25 and 35, respectively.

Serum lipid peroxides were significantly lower in pups from the HFDL litter compared to those from the HFD group at P25 and P35 (21⋅6 % lower, *P* = 0⋅005; 30⋅4 % lower, *P* = 0⋅003, respectively) (Fig. 3(a)). No significant differences in AC were observed between groups at either time point (Fig. 3(b)).

#### Circulating adipokines, glucose and inflammatory cytokines

**Fig. 4.**
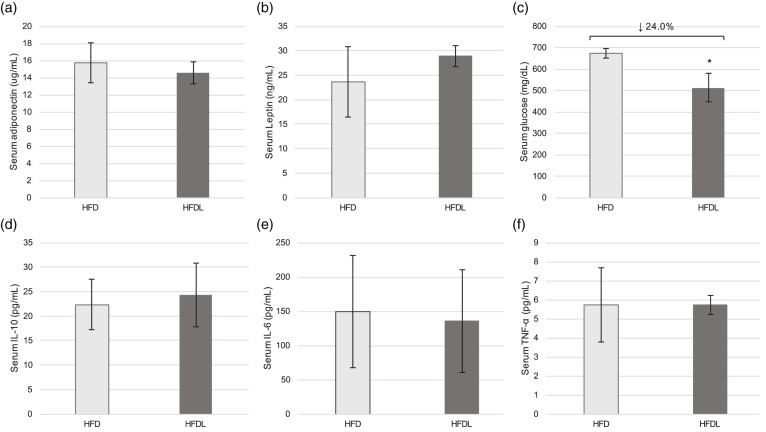
Serum metabolic outcomes in offspring. Serum adipokines (a, b), glucose (c), and inflammatory markers (d–f) at P25 in offspring of Sprague-Dawley mothers fed a high-fat diet with or without lycopene supplementation. Bars represent the mean ± standard deviation. *Indicates *P* < 0⋅05 between-group difference. HFD, high-fat purified diet (50 %), HFDL, high-fat purified diet (50 %) with 1 % lycopene supplementation; IL-6 and IL-10, interleukin-6 and -10, respectively; P25, postnatal day 25; TNF-α, tumour necrosis factor-α.

Serum adiponectin and leptin did not differ significantly between groups despite the aforementioned modifications in AT development (Fig. 4(a) and (b)). No significant differences were observed in IL-10, IL-6 or TNF-α between groups (Fig. 4(d)–(f)). However, glucose was 24 % lower (*P* = 0⋅004) in pups reared by mothers consuming the HFDL at P25 (Fig. 4(c)). Significant correlations between glucose and WAT mass (*r* 0⋅771, *P* = 0⋅025), BAT mass (*r* −0⋅766, *P* = 0⋅027) and lipid peroxides (*r* 0⋅827, *P* = 0⋅011) were also observed at P25.

## Discussion

The present study aimed to determine the influence of lycopene supplementation on AT development and obesity-associated metabolic outcomes in the neonate and weanling offspring of Sprague-Dawley rat mothers fed an HFD. In short, results demonstrated that lycopene supplementation suppressed WAT accrual while augmenting BAT development in offspring, as well as improved serum oxidative stress and glucose levels. To our knowledge, this is the first study demonstrating the protective effects of lycopene supplementation, in the context of a high-fat diet, on AT development and metabolic health during critical periods of early development.

Previous research has established the consequences of maternal obesity such that excess adiposity during gestation may induce cellular and developmental alterations in offspring. This has been attributed to the phenomenon known as metabolic programming^(2,27)^. Such alterations deleteriously influence AT differentiation with subsequent disruptions of metabolic homeostasis^(3,28)^. As such, maternal obesity and excessive pregnancy weight gain may contribute to long-term cardiometabolic disease risk in offspring. Acknowledging the potential for dietary modifications to reverse the detrimental outcomes of metabolic programming, the National Institutes of Health (NIH) highlights this research area as a strategic goal in its *2020–2030 Strategic Plan for NIH Nutrition Research*^(29)^. In the present study, maternal lycopene supplementation was initiated immediately following delivery of pups while maintaining a high-fat diet. This was designed to resemble the addition of lycopene-rich foods to the diet and, thus, not predicated on a major behaviour change. Such dietary modifications have an increased likelihood of sustained integration into a person's daily life^(30)^. This method of maternal supplementation resulted in offspring indirectly receiving lycopene via the lactation diet until P25. Since indirect lycopene supplementation on AT development and metabolic health of neonates and weanling offspring has not been previously investigated, it is important to evaluate maternal-offspring lycopene transfer and its influence on the aforementioned outcomes. In the present study, it was apparent that lycopene was transmitted into the circulation of the HFDL group as evident by confirmed lycopene peaks at 450 nm. Similar peaks were not observed in serum of offspring not receiving lycopene supplementation.

### Effects of lycopene supplementation on AT development in offspring

Two primary types of AT stores exist: WAT and BAT. In addition to serving as the main storage site of triglycerides, the endocrine nature of WAT promotes systemic metabolic abnormalities^(4)^. BAT is in stark contrast to WAT such that its dense mitochondrial make-up and expression of UCP1 enables it to dissipate energy in the form of heat through adaptive thermogenesis^(6)^. As previously referenced, maternal obesity influences the development of AT depots through various mechanisms^(2,28)^. Previous studies in which offspring were subjected to an obesogenic gestational environment led to a significantly greater BW and visceral WAT accumulation at birth and maintained through the weaning period^(31,32)^. The present study supports previous findings such that pups reared by mothers consuming an HFD exhibited significantly greater BW and visceral WAT throughout early development compared to those in the NFD group. Supplementation with lycopene, however, resulted in significantly lower WAT depots in the offspring, as well as a reduction in AI. Similar anti-adiposity effects of lycopene have been observed in adult animal models in which an HFD (ranging from 45 to 60 % kcal from fat) was implemented^(18,33,34)^. Although the role of lycopene in WAT development has yet to be fully elucidated, it is hypothesised that these effects are due in part to alterations in the peroxisome proliferator-activated receptor gamma (PPAR-γ) expression in white adipocytes or through the process of autophagy within WAT^(33–35)^. While the molecular mechanisms underpinning changes in WAT accrual with lycopene supplementation require further investigation, results from the present study demonstrate the physiological influence of lycopene in preventing WAT accrual in offspring of mothers consuming an HFD.

Acknowledging the inverse relationship between BAT stores and body fat gain in early life, nutritional strategies to optimise BAT development and its preservation in the neonate are warranted^(9)^. Certain carotenoids and their respective conversion products have been shown to metabolically activate adaptive thermogenesis^(11,12)^. The promoter region of the UCP1 gene contains non-canonical retinoic acid-response elements (RARE), as well as PPAR response elements that are mediated by retinoic acid receptors (RARs) and retinoid X receptors^(27,36,37)^. Results from *in vitro* models have demonstrated the ability of retinoic acid, a vitamin A derivative, to increase UCP1 mRNA expression in brown adipocytes through its interaction with RAR-α to activate certain RAREs in the UCP1 gene^(12)^. Similar *in vitro* results have been obtained for β-carotene, α-carotene and lutein^(11)^. Previous research has reported that lycopene and its metabolites also induce RAR activation in both *in vitro* and *in vivo* animal models, thus potentially influencing BAT development and activation^(38,39)^. Additionally, lycopene may impact BAT development through its reported upregulation of PPAR-γ, which can induce expression of other thermogenic genes, namely UCP1^(34,40)^. In the present study, lycopene supplementation resulted in a 65 % significant increase in BAT mass at P25 with similar patterns persisting into P35, albeit non-significant. Interestingly, no significant differences in mRNA expression of UCP1 in BAT were observed between groups. This is in contrast to a recent study conducted by Zhu *et al.*^(34)^ in which a 45 % HFD with lycopene supplementation (15 mg/kg/d) for 10 weeks markedly increased the number of brown adipocytes in interscapular BAT with corresponding upregulation of UCP1. Similarly, a separate study utilising 3-month-old mice reported a significant increase in UCP1 expression following 10 weeks of a 45 % HFD with 0⋅03 % lycopene supplementation^(35)^. Discrepancies in study results may stem from the method of supplementation (i.e. maternal transfer of lycopene *v*. direct consumption), dosage of lycopene, as well as the timeline of supplementation. In summary, results from the present study suggest that although maternal lycopene supplementation increased BAT mass in the neonate and weaning periods, it was unable to upregulate UCP1 mRNA expression to enhance the functionality of BAT^(5)^. It is possible that despite an increased BAT mass, there were either fewer or smaller mitochondria per brown adipocyte^(41)^.

### Effects of lycopene supplementation on obesity-associated metabolic outcomes in offspring

Maternal obesity has been associated with increased circulating oxidative stress in newborns^(42)^. While the antioxidant properties of lycopene have been studied extensively in adults, its impact on redox status in neonates has yet to be evaluated. Although serum lycopene remained above the level of detection but below the level of quantitation, it was apparent that this carotenoid was transmitted into circulation in the HFDL pups. This transmittance likely conferred systemic benefits, as the HFDL group exhibited significantly reduced serum lipid peroxides compared to the HFD only pups at P25 and P35. A substantial difference in AC was not observed between the two groups; however, it is plausible that lycopene was utilised as an antioxidant to directly quench free radicals and/or to upregulate endogenous antioxidant expression and indirectly attenuate levels of oxidative stress^(43)^.

Maternal lycopene supplementation in the HFD group reduced serum glucose of the offspring by approximately 24 % at P25. Oxidative stress has been implicated in alterations of glucose dynamics. Increased concentrations of reactive oxygen species activate stress-induced pathways, including nuclear factor κ B and mitogen-activated protein kinase, which diminishes the insulin response and subsequent glucose uptake^(44,45)^. As such, the improvements in circulating measures of oxidative stress in the offspring likely conferred additional benefits beyond redox status, including glucose regulation. In support of this, a significant correlation was observed between serum glucose and lipid peroxide concentrations at P25 (*r* 0⋅827, *P* = 0⋅011). Similar improvements in glucose levels have been obtained in studies implementing an HFD supplemented with lycopene as well as tomato-based powders^(18,34)^.

Adipokines, such as leptin and adiponectin, influence metabolic homeostasis, as well as overall cardiometabolic risk through their anti-inflammatory and insulin-sensitising properties, among others^(46)^. A hallmark of AT dysfunction is the alteration of adipokine secretion such that circulating leptin concentrations are increased with a concomitant reduction in adiponectin. In the present study, the HFDL group exhibited a significantly lower WAT mass at P25, yet there were no significant modifications in leptin or adiponectin secretion at this time point. An absence in adipokine alterations despite modifications in AT accrual may stem from a distinction in the secretion of adipokines between the neonate and adult. For example, adiponectin is secreted by various tissues in the foetus/neonate (i.e. placenta, skeletal muscle, small intestine and AT), whereas it is solely secreted by WAT in adult human subjects^(47,48)^. The systemic production of adiponectin during early life could obscure changes related to AT function. Additionally, it is plausible that the distinction in adiposity at the time of adipokine assessment was not sufficient to induce physiological changes. Previous lycopene supplementation research in adult models has been demonstrated to improve mRNA expression in WAT and/or circulating levels of adipokines^(18,33,35,49)^. Likewise, no differences in inflammatory cytokines were appreciated between groups in the present study despite the well-established anti-inflammatory properties of lycopene^(18,33,50)^. It should be noted that the assessment of the aforementioned metabolic measures took place at P25 only due to a limited sample. Up until this time point, offspring had primarily received indirect lycopene supplementation through maternal transfer. Therefore, it is possible that this indirect route of supplementation resulted in a weaker influence of lycopene on the metabolic perturbations of obesity, beyond redox status. Thus, an extended timeframe of assessment that includes the offspring directly ingesting lycopene-supplemented diets may reveal stronger modifications in metabolic outcomes.

### Strengths and limitations

Through the investigation of maternal lycopene supplementation paired with a high-fat diet, this exploratory study advances the current understanding of carotenoid supplementation on adiposity and related metabolic outcomes during critical periods of development. Results are strengthened by the use of purified diets to limit confounding variables. In this exploratory pilot study, multiple time points were evaluated throughout this rapid period of development while also satisfying the *a priori* power calculation^(51)^. P14, P25 and P35 represent the neonatal, weanling and peri-adolescent phases of rats, respectively, thus capturing the dynamic changes in AT and metabolic homeostasis of early life^(52)^. Furthermore, this is the first study to evaluate the influence of lycopene supplementation on clinically relevant alterations in offspring subjected to an obesogenic gestational environment. In light of results reported herein, future research investigating the molecular mechanisms underpinning lycopene supplementation and AT development in the neonate is warranted, as well as evaluating the potential ‘beiging’ effect of lycopene on WAT.

Despite study strengths, it is not without inherent limitations. Given the developmental timeframe under evaluation, the tissue and serum collected from pups were relatively low at early necropsy time points, and consequently, certain outcome measures could not be assessed at all intervals. This exploratory study was also designed to represent the effects of obese mothers beginning a dietary lycopene intervention after giving birth to offspring. Nevertheless, an expanded timeframe of investigation with the supplementation of lycopene beginning prior to gestation is needed to determine whether improvements in adiposity and obesity-associated metabolic perturbations are amplified. Lastly, the dose in the present study was selected to maximise maternal lycopene consumption and ensure sufficient transfer to offspring for the evaluation of adiposity and cardiometabolic outcomes. While extrapolation to a human equivalent dose may not be reasonably attained exclusively through the diet, it could potentially be met through supplementation. Acknowledging that a link between maternal lycopene supplementation and cardiometabolic outcomes, notably BAT development, has been established in the present study, future research evaluating this relationship with varying doses of lycopene is warranted.

Taken collectively, results suggest that maternal lycopene supplementation has the potential to suppress WAT accrual while augmenting BAT development in offspring, as well as to improve certain measures of obesity-associated metabolic outcomes.
